# Primary Kaposi’s sarcoma of the nasal cavity: a case report and review of the literature

**DOI:** 10.1186/s13569-016-0044-4

**Published:** 2016-03-17

**Authors:** Karima Mouden, Mouna Khmou, Saida Loughmari, Afaf Semmar, Hanan El Kacemi, Basma El Khannoussi, Tayeb Kebdani, Sanaa Elmajjaoui, Noureddine Benjaafar

**Affiliations:** Department of Radiotherapy, National Institute of Oncology, University Mohammed V, Rabat, Morocco; Department of Pathology, National Institute of Oncology, Rabat, Morocco

**Keywords:** Kaposi sarcoma, Nasal cavity, Adequate immune system, Chemotherapy

## Abstract

**Background:**

Kaposi sarcoma is a neoplastic vascular disorder. It usually present on the skin of the upper and lower extremities, rarely in the mucosa of the head and neck. The most common sites reported are within the oral cavity, particularly on the palate. Other mucosal sites are rare. We present an unusual case where the primary manifestation of the Kaposi’s sarcoma was in the nasal mucosa.

**Case presentation:**

A 56-year-old female of Mediterranean descent presented with a 1 year history of swelling on the left side of her nose, nasal obstruction and occasional minor epistaxes. Physical examination showed a firm and bulging polypoid mass which filled the left nasal cavity without cutaneous lesions.

Computed tomography (CT) demonstrated a tumor, measuring 77 mm in diameter, occupying the left nasal cavity causing erosion of nasal septum and extending posteriorly to the left choana and nasopharynx. There was bilateral cervical lymphadenopathy. Patient treated with chemotherapy alone. She was in a complete response after the first cycle. The patient received no further treatment. She needs a regular medical checkups that include a review of a patient’s medical history and a complete physical exam. She is in excellent local control over 12 months.

**Conclusions:**

A review of the literature revealed that only seven cases of primary Kaposi sarcoma of the nasal cavity have previously been published and only two of them presented in a patient not associated with the acquired immunodeficiency syndrome. Here, we report the third case where the primary manifestation of the Kaposi sarcoma was in the nasal cavity in a patient with an adequate immune system.

## Background

Kaposi’s sarcoma (KS) is a low-grade vascular tumor associated with infection with human herpesvirus 8 (HHV-8), also known as the KS-associated herpesvirus (KSHV) [1, 2].

The disease is named after Moritz Kaposi, a Hungarian dermatologist on the faculty of the University of Vienna, who first described the entity in 1872 as “idiopathic multiple pigmented sarcoma of the skin” [3]. The most recurrently affected sites are the skin and mucous membrane [4, 5], however, it can be found in other parts of the body: lungs, stomach, intestine [6–8], etc.

The KS of the head and neck usually involves the mucous membranes of the oral cavity, but it can be found in the pharynx, larynx, and tonsillar [9–18]. Isolated nasal location is extremely rare [19–23].

A review of the literature revealed that there are a few reported cases of KS involving the head and neck region. In fact, only seven had primary presentation in the nasal cavity and two of them were not AIDS related [20, 21, 24–26].

Here, we present a case of KS of the left nasal cavity in HIV negative patient, treated by chemotherapy only.

## Case report

A 56-year-old female of Mediterranean descent presented with a 1 year history of swelling on the left side of her nose, nasal obstruction and occasional minor epistaxes. Physical examination showed a firm and bulging polypoid mass which filled the left nasal cavity without cutaneous lesions.

**Fig. 1:**
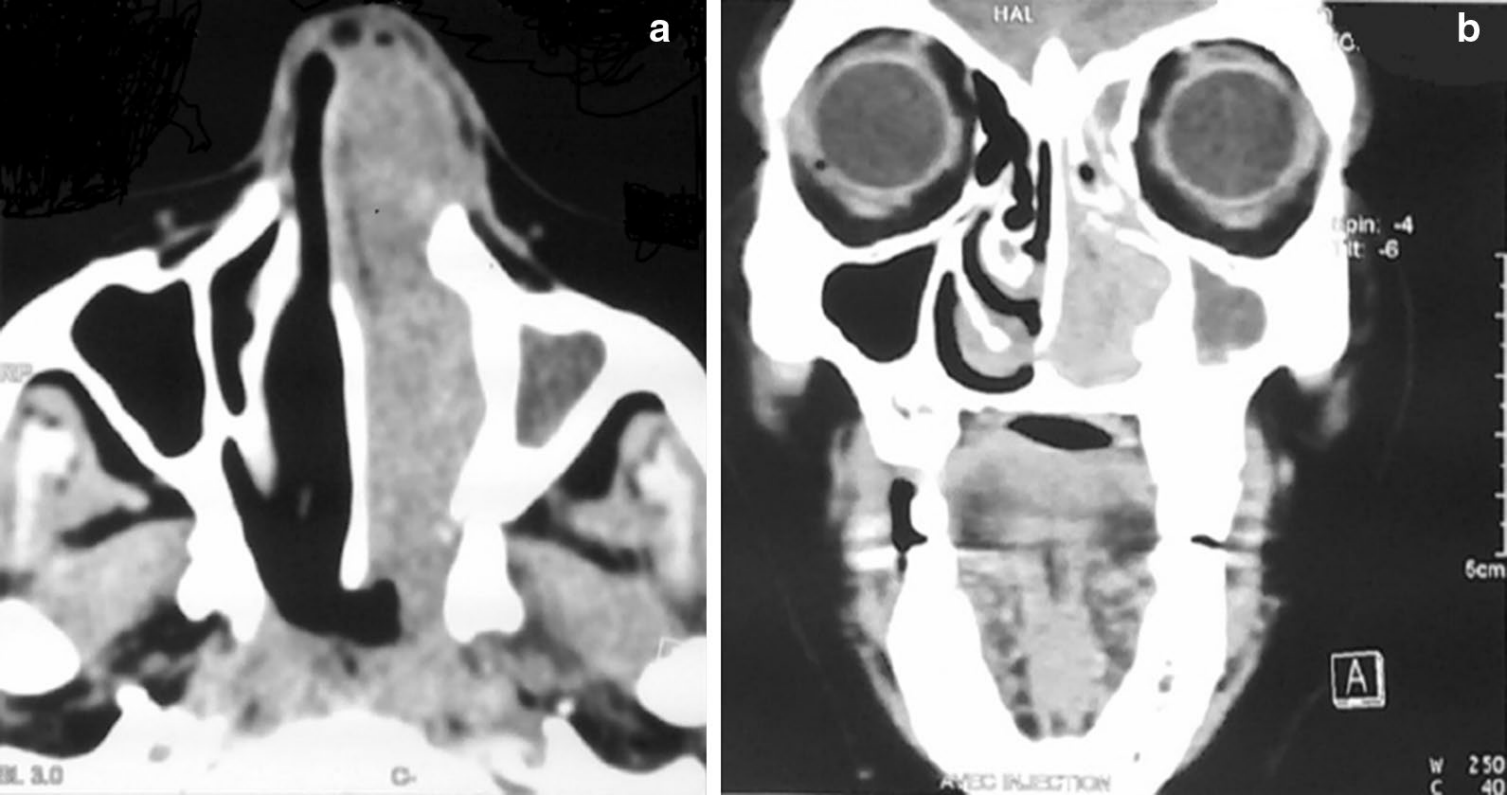
**a** Axial CT, **b** Coronal CT: demonstrated a tumor occupying the left nasal cavity causing erosion of nasal septum and extending posteriorly to the left choana and nasopharynx

Computed tomography (CT) (Fig. 1) demonstrated a tumor, measuring 77 mm in diameter, occupying the left nasal cavity causing erosion of nasal septum and extending posteriorly to the left choana and nasopharynx. There was bilateral cervical lymphadenopathy.

A biopsy specimen was obtained in the nasal cavity. Initially, the diagnosis of angiosarcoma was made. After multidisciplinary consultation meeting, a second pathological opinion was required. Original slides examination revealed a submucosal proliferation composed of intersecting ill-defined fascicles, of spindled cells with mild to moderate nuclear atypia. Slit-like spaces containing red blood cells were noted. Intracytoplasmic and variably sized hyaline globules were seen in some tumor cells. Immunohistochemical study revealed a strong expression of CD31, CD34, and vimentin. Tumor cells showed negative staining for AE1/AE3, CK5/6, HMB-45, and melan-A. As tissue was depleted in the paraffin block, we were unable to perform further immunohistochemical testing, using HHV 8 antibody. A final diagnosis of Kaposi’s sarcoma was rendered.

A chest CT did not showed metastases in the lungs.

Decision in multidisciplinary consultation meeting is to instaure neoadjuvant chemotherapy because surgical excision is not feasible. Then an evaluation must be performed. Chemotherapy drugs ifosfamide (IFO) and doxorubicin (DOXO) was started on December 22, 2014. The detailed treatment regimen was as follows: IFO 1800 mg/m2 i.v. d1-5; mesna dose is 100 % of the IFO dose (3 g) i.v. d1-5 and DOXO 60 mg/m2 d1.

The cycle was repeated every 21 days. Physical examination after the first cycle of treatment showed a complete response. To keep the clinical response, the patient continued chemotherapy until four cycles. The fourth cycle date April 27, 2015.

**Fig. 2:**
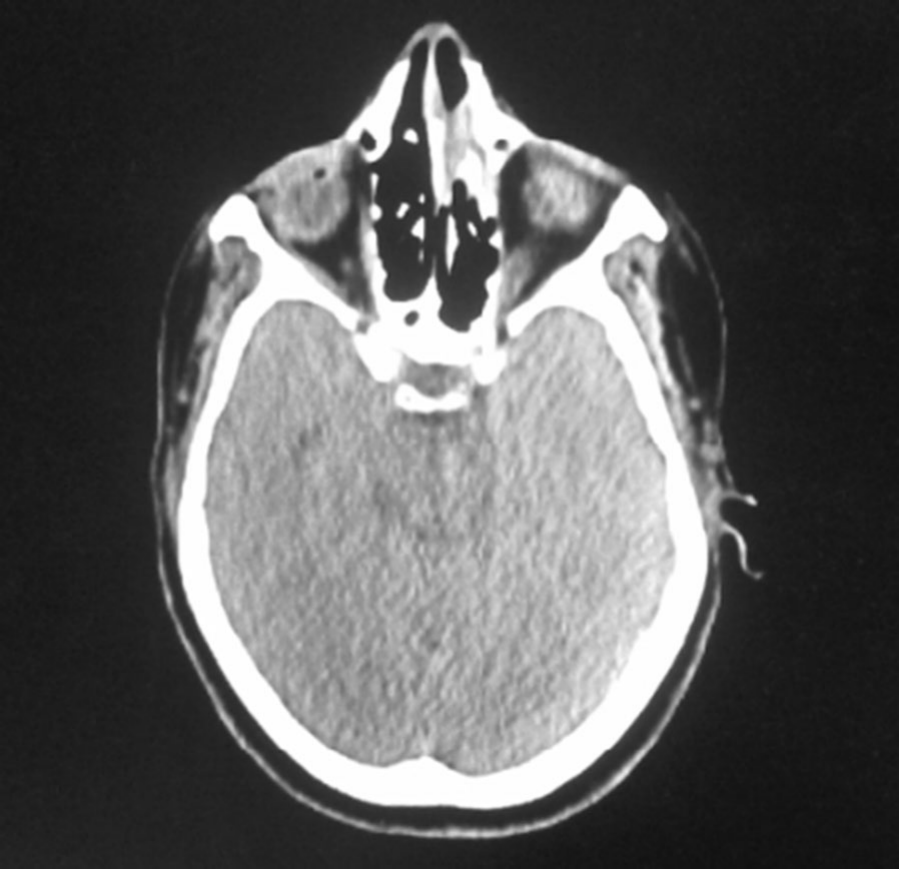
Axial computed tomography (CT) of evaluation showed a near complete response in the nasal cavity

Computed tomography (CT) of evaluation showed a near complete response in the nasal cavity (Fig. 2).

The patient received no further treatment. She needs a regular medical checkups that include a review of a patient’s medical history and a complete physical exam. She is in excellent local control over 12 months.

## Discussion

Sinonasal cancers, which account for 3 % of all head and neck cancers, have their origin in the nasal cavity in 20–30 % of cases [27]. It is relatively uncommon [28–30], with an incidence of 0.75 per 100,000 in the United States. This is a heterogeneous group of uncommon neoplasms including adenocarcinomas, malignant melanomas and non-Hodgkin’s lymphomas, followed in frequency by sarcomas, esthesioneuroblastomas, hemangiopericytomas, plasmacytomas, and anaplastic carcinomas [31–34].

KS is an important opportunistic disease, which frequently occurs in HIV-positive patients. The incidence of KS involving head and neck was reported to be as high as 40–67 %, in which the cutaneous lesions predominate [25, 26].

However, the involvement of this region is only 14 % of patients with HIV-negative [24, 35–41].

Here, we reported a case de Kaposi’s sarcoma of the nasal cavity in patient with HIV-negative.

KS involvement has been observed in almost all visceral sites, including lymph nodes, liver, pancreas, heart, the testes, bone marrow, bone, skeletal muscle and mucosa of head and neck [42, 43].

The most frequent sites of noncutaneous disease are the oral cavity, gastrointestinal tract, and respiratory system [44–48].

There is a few cases of the Kaposi’s sarcoma in the nasal mucosa [25, 26, 49].

In a review of the literature, seven cases of the Kaposi’s sarcoma in the nasal mucosa were reported.

Gnnep et al. discussed nine cases of KS arising in the head and neck region. One of them was in the nose-mucocutaneous junction with HIV-negative, treated by surgical excision and relapsed at 9 months [24].

The second case of the KS in the nasal cavity with an adequate immune system was reported by Ioannis Venizelos et al. It was a case of a 59 year-old woman who presented a tumor arising from the left nasal septum which was excised. The patient received no further treatment and 4 years later, she is in excellent condition [21].

Three patients with HIV-positive who had KS of the nasal cavity, were treated by surgical excision. One of them relapsed at 2 months [20, 25, 26].

Patients with tumors of the nasal cavity usually present with a history of chronic sinus congestion and recurrent nasal obstruction. In our case, there was no ulceration of the overlying squamous epithelium and the patient presented with swelling on the left side of her nose, nasal obstruction and occasional minor epistaxes.

Various clinicopathologic forms of Kaposi’s sarcoma have been described, which often have overlapping features (patch, plaque, nodular, lymphadenopathic, infiltrative, florid, telangiectatic, ecchymotic, keloidal, angiomatous, inflammatory, pleomorphic, lymphangiomatous, and generalized lymphedema). In the earliest stage of Kaposi sarcoma, a flat lesion made of miniature vessels surrounding larger ectatic vessels is noted [50, 51].

In the more advanced stage of the disease, a discernible but relatively bland spindle cell component, centered around the proliferating vascular channels, can be described. Coalescent foci of spindle cells produce the classic nodular lesions of Kaposi sarcoma [51].

The spindle cells are separated by slit and sieve-like spaces containing erythrocytes and vascular channels. Lymphocytes and plasma cells, hemosiderin deposits, and dilated vessels are commonly seen at the periphery. Both intracellular and extracellular hyaline globules may be identified [52].

The tumor cells are strongly immunoreactive for CD31, CD34, factor VIII–related antigen, and Latent nuclear antigen-1 of HHV-8 (Kaposi sarcoma-associated herpes virus. They are variably immunoreactive for smooth muscle actin and are negative for cytokeratins (AE1/AE3 and CAM 5.2). The nuclear positivity using the LANA-1 antibody confirms the diagnosis [50, 51, 53].

The histologic differential diagnoses of Kaposi’s sarcoma include angiosarcoma, fibrosarcoma, arteriovenous malformations, and spindle cell hemangioendothelioma.

Clinicopathological features, mainly immunohistochemical studies, help by excluding these differential diagnoses, but some cases with unusual findings can pose a challenge to general pathologists [50, 52, 53].

The major goals of treatment are symptom palliation, prevention of disease progression, and shrinkage of tumor to alleviate edema, organ compromise, and psychological stress [54].

For HIV-positive patients who develop either limited or advanced Kaposi sarcoma (KS), it is recommended that treatment includes highly active antiretroviral therapy (ART).

Local treatment modalities are useful for cosmesis or the management of symptomatic bulky KS lesions, but they do not prevent the development of new lesions in untreated areas. The most widely used local treatment approaches include:

Intralesional chemotherapy with vinblastine, radiation therapy and topical alitretinoin.

Indications for systemic chemotherapy include extensive cutaneous disease, symptomatic visceral involvement, and cutaneous KS that is unresponsive to local therapy.

When chemotherapy is indicated, treatment with pegylated liposomal doxorubicin or liposomal daunorubicin is generally recommended as the first-line treatment for KS [55]. Other agents that have been used include paclitaxel, bleomycin, vinblastine, vincristine, and etoposide [56].

In our case, chemotherapy seems to be effective, since 8 months later the patient is in excellent condition without evidence of local recurrence or metastasis both clinically and radiologically.

## Conclusions

Primary Kaposi sarcoma of the nasal cavity is a very rare entity. Our case represents the third reported case not associated with AIDS. Chemotherapy with doxorubicin seems to be effective with in excellent response.

## Consent

Written informed consent was obtained from the patient for publication of this case report and any accompanying images.
